# Rectal Drainage of Pelvic Appendicular Abscesses

**Published:** 1908-09-26

**Authors:** 


### RECTAL DRAINAGE OF PELVIC APPENDICULAR ABSCESSES.

It is a well-known aphorism that no case of suspected appendicitis has been properly investigated if a rectal examination has been omitted; the information that the finger in the rectum often conveys to one's brain is again and again of the greatest value in diagnosis. It is surprising that the same information is not more often utilised in the treatment of the case. When one comes to think of it, it is almost marvellous that appendicular abscesses drain as well as they do when operated upon by the abdominal route; at least part of the pus has to escape by a path along which gravity is opposed to any mechanical outflowing. The pus has to well out or to be sucked out by gauze wicks, or else it has to be driven out by the production of granulations beneath it. There are, of course, appendicular abscesses which lie entirely above the level of the ordinary incision for appendicitis; but there are a great many more that are at least partly below that level; and there are some that are entirely below it, being located in the pelvis, and recognisable there by rectal examination. It is with these that we are concerned in the present note.

If the best way to drain an abscess is to open it freely in its most dependent part, then there are many appendicular abscesses which should be opened per rectum. Cases of appendicitis in which pelvic collections of pus occur have been divided by Mr. Bilton Pollard, of University College Hospital, into two groups, namely:—

1. Cases of the following type: An appendicular abscess has formed in the right iliac fossa, above the brim of the true pelvis. At the time of the operation no collection of pus can be detected in the pelvis by rectal or vaginal examination. The temperature falls, and all goes well for a few days. The temperature then goes up again, and the pulse increases in frequency. The drainage of the abscess already opened appears to be quite satisfactory, and consequently a further examination of the pelvis by rectal or vaginal examination is made, and the relapse is explained by the presence of a collection of pus in the basin of the true pelvis.

2. Cases of the following type: The illness has begun in the manner characteristic of appendicitis. The temperature continues high and the pulse rapid, and so perhaps a week goes by. No mass is felt by abdominal palpation, but as the case is not progressing satisfactorily, a surgeon is called in, and his routine rectal examination discovers a pelvic swelling bulging the anterior rectal wall, either alone or in association with resistance or swelling behind the right rectus muscle.

It might have been objected that the rectum was so full of micro-organisms of all kinds that to place it in direct communication with a pelvic abscess in which, presumably, only one or at most two varieties of organism—the *bacillus coli communis* and some other—would be present, might be dangerous and bad; as a matter of fact, however, the danger is theoretical only. Many a case ends by the spontaneous opening of the abscess into the rectum, recovery taking place uneventfully. Many another case has had the abscess opened surgically per rectum, with far better chances of a good result than if the abscess is left to burst by itself; for the surgical opening is made clean and large, whereas the spontaneous opening is devious, small, and ulcerous. The possibility of danger from contamination from the rectum is surprisingly small, and it is difficult to understand why surgeons avail themselves of the rectal route so seldom when cases present themselves with the abscess almost pointing in that direction. Gynæcologists open and drain pelvic abscesses per vaginam, seldom dreaming of opening the abdomen in such cases if doing so can be avoided. Surgeons to a great extent have hitherto acted upon the converse principle—preferring laparotomy, whenever possible, to operation by any other route in cases of appendicitis.

Just now there is a good deal of discussion as to whether the vermiform appendix should not always be looked for and removed whenever possible in every case of appendicular abscess at the time the abscess itself is operated upon. The ground upon which some surgeons have firmly advocated removing the appendix at the same time as opening the abscess is that cases in which the appendix is left are very apt to have recurrence of the abscess later on, whereas such recurrence does not take place if the appendix has been removed. It is clearly impossible to remove the vermiform appendix per rectum; hence any surgeon who is a very strong supporter of the view that its stump should on no account be left would naturally be averse to the rectal route in draining an appendicular abscess. The opinion which seems to be supported by the majority is that, in dealing with a well localised appendicular abscess, whatever its situation, it is not wise to do more than is absolutely necessary at the moment to provide for the efficient evacuation and drainage of the pus out of the abscess cavity. If the appendix is readily found, well and good: let it be ligatured off and removed forthwith; if, on the other hand, it requires to be searched for, it is much better to leave it entirely alone. Its complete removal involves the breaking down of limiting adhesions. The latter protect the patient from infection of the general peritoneal cavity, and the less they are disturbed the better. The patient is better off if he suffers from a recurrent abscess later on than if he develops general peritonitis now.

Neither the danger of further infection, therefore, nor the impossibility of removing the appendix at the same time constitutes a real argument against the rectal opening of such appendicular abscesses as can be dealt with that way. The advantages of the method are clear. The pus is found and let out by a simple incision which is a very different thing to the difficult operation of finding one's way by the abdominal route between coils of matted bowel, and possibly adhesions so firm and obliterating that separation of coils of intestine from one another may be attended with grave risk of injuring the healthy bowel walls. The abscess drains excellently from its most dependent part. There is no scar in the patient's abdominal wall, weak because the site of former sepsis, and abdominal hernia is avoided.

